# Supplementary material for: From sequence to enzyme mechanism using multi-label machine learning
Source: BMC Bioinformatics. 2014 May 19;15:150. doi: 10.1186/1471-2105-15-150 (PMC4229970; doi:10.1186/1471-2105-15-150)
Supplement: Additional file 2 — Java code of ml2db. Additional file ml2db_code.tar.gz contains the Java source code to run the multi-label machine learning experiments and save the results to database. The code’s Javadoc is included. [file 1471-2105-15-150-S2.zip › additional file 2/ml2db/utils/doc/uk/ac/standrews/utils/main/stats/package-summary.html]

uk.ac.standrews.utils.main.stats


JavaScript is disabled on your browser.


- Overview
- Package
- Class
- Use
- Tree
- Deprecated
- Index
- Help

- Prev Package
- Next Package

- Frames
- No Frames

- All Classes

# Package uk.ac.standrews.utils.main.stats

- Class Summary

  | Class | Description |
  | --- | --- |
  |  |  |
  | --- | --- |
  | PseudoTruncatedPareto | A truncated pareto distribution which stores a big lists of power-law extracted random numbers. |
  | PseudoTruncatedParetoSingleton | Class |
  | StatUtils | Utilities to print simple histograms, percent of frequency etc |
  | TruncatedPareto | A utility class to extract from a truncated Pareto (power-law, long-tail) distribution. |
  | UniformRandomSingleton | Class |
  | UniformRandomUtils | Utilities for extracting with uniform random distribution |

- Overview
- Package
- Class
- Use
- Tree
- Deprecated
- Index
- Help

- Prev Package
- Next Package

- Frames
- No Frames

- All Classes
